# Development of simple and rapid assay to detect viral RNA of tick-borne encephalitis virus by reverse transcription-loop-mediated isothermal amplification

**DOI:** 10.1186/1743-422X-10-68

**Published:** 2013-03-04

**Authors:** Daisuke Hayasaka, Kotaro Aoki, Kouichi Morita

**Affiliations:** 1 Department of Virology, Institute of Tropical Medicine, Global COE Program, Leading Graduate School Program, Nagasaki University, 852-8523, Nagasaki, Japan

**Keywords:** TBEV, RT-LAMP, Far-eastern subtype, Siberian subtype, European subtype

## Abstract

**Background:**

Tick-borne encephalitis virus (TBEV) is a causative agent of acute central nervous system disease in humans. It has three subtypes, far eastern (FE), Siberian (Sib) and European (Eu) subtypes, which are distributed over a wide area of Europe and Asia. The objective of this study was to develop a simple and rapid assay for the detection of TBEV RNA by using reverse-transcriptase loop-mediated isothermal amplification (RT-LAMP) method that can differentiate the three subtypes of TBEV and can be used for clinical diagnosis and epidemiological study.

**Methods:**

Primers for TBEV-specific and subtype-specific RT-LAMP assay were designed to target the consensus sequence in NS1 of all subtypes and the consensus sequence in the E gene of each subtype, respectiveluy. *In vitro* transcribed RNA of Oshima strain that belongs to FE subtype was serially diluted and used to examine the sensitivity of the assay. Cross-reactivity of subtype-specific RT-LAMP assay was tested by using the RNA of Oshima and Sofjin (FE), IR-99 (Sib) and Hochosterwitz (Eu) strains. RNA extracted from the mixtures of TBEV and ticks, and of TBEV and human blood, and the mouse tissues infected with TBEV, were evaluated in the assay. Positive amplification was observed by real-time monitoring of turbidity and by visual detection of color change.

**Results:**

The sensitivity of TBEV-specific RT-LAMP assay was 10^2^ copies of target RNA per reaction volume. FE-specific RT-LAMP assay amplified viral genes of Oshima and Sofjin strains but not of IR-99 and Hochosterwitz strains, and of Japanese encephalitis virus. RT-LAMP assay for Sib and for Eu specifically amplified viral genes of IR-99 and Hochosterwitz strains, respectively. We also showed that tick or human blood extract did not inhibit the amplification of viral gene during the assay. Furthermore, we confirmed that the TBEV RT-LAMP could detect virus RNA from peripheral and central nervous system tissues of laboratory mice infected with TBEV.

**Conclusion:**

TBEV RT-LAMP assay offers a sensitive, specific, rapid and easy-to-handle method for the detection of TBEV RNA in tick samples and this may be applied in the clinical samples collected from TBE-suspected patients.

## Background

Tick-borne encephalitis virus (TBEV), which belongs to the genus *Flavivirus* in the family *Flaviviridae*, is a causative agent of acute central nervous system (CNS) disease in humans and animals [1,2]. Its genomic RNA consists of one open reading frame and encodes three structural proteins (C, M and E) and seven nonstructural proteins (NS1, NS2A, NS2B, NS3, NS4A, NS4B and NS5) [3]. TBEV is transmitted by *Ixodes* tick species and rodents in nature, and infects humans through the bite of an infected tick [1,2,4]. It is distributed over a wide area of Europe and Asia, and is geographically and genetically divided into three subtypes, far eastern (FE), Siberian (Sib) and European (Eu) [5-7]. The vector of FE and Sib subtypes is *Ixodes persulcatus* and that of Eu subtype is *Ixodes ricinus*[7].

It has been suggested that the FE subtype is associated with more severe disease than the other subtypes, although the morbidity rate for the disease is different in Europe and Russia, perhaps at least partly due to a selective registration mostly of severe cases in these areas [3,7,8]. This raises concern on the risk of tick-borne encephalitis (TBE) in each country or endemic area, where different TBEV subtypes may be distributed. Therefore, surveillance on the prevalence of TBEV in ticks or serological monitoring of rodent is required to assess the risk of human infection caused by this virus in the endemic areas [9-11].

In human cases, TBE characteristically takes a biphasic course involving an acute febrile illness, and a period of apparent recovery, followed by a neurological syndrome [2,12]. The neurological symptoms include headache, meningitis, meningoencephalitis and meningoencephalomyelitis, the latter being observed in the most severe cases. When death happens, it is usually within 5 to 7 days from the onset of neurological signs [1]. However, such clinical features are not unique to TBE, thus, laboratory diagnosis is required to distinguish it from other neurological disorders [12,13].

For the surveillance on the prevalence of TBEV in ticks and other animals, or for clinical diagnosis, molecular techniques based on genomic detections such as reverse-transcriptase polymerase chain reaction (RT-PCR) and real-time RT-PCR (rRT-PCR) have been developed [10,11,14-19]. However, RT-PCR method requires specific equipment such as thermal cycler, electrophoresis tank and UV illuminator. rRT-PCR has some advantages over conventional RT-PCR and allows high magnitude of amplification, but it requires high-precision instruments for the amplification and a complicated method for the detection of amplified products.

The loop-mediated isothermal amplification (LAMP) assay is a rapid, accurate, and cost-effective diagnostic method to amplify the DNA under isothermal conditions at 60°C to 65°C [20,21]. The one-step RT-LAMP assay requires six primers: two inner primers and two outer primers that define the target region, and two loop primers for increasing the sensitivity of the assay. The final DNA products of this assay consist of multiple loops with several repeats of the target sequence, and can be detected by agarose-gel electrophoresis, real-time monitoring of turbidity or visualization of color change. This method has been used to detect a number of RNA viruses including some flaviviruses [22-26]. Its advantage of over RT-PCR and rRT-PCR is that it makes use of inexpensive and simple equipment such as a heating block or a water bath. Thus, the LAMP method has a potential for use as a simple tool for rapid laboratory confirmation of infectious disease even in a resource-limiting setting.

In this study, we report the development of one-step RT-LAMP assay specific to TBEV and to each of the three subtypes. This assay will be useful in the surveillance of the prevalence of TBEV in ticks and other animals in the field and in the diagnosis of TBEV infection in humans.

## Results

### Sensitivity of TBEV-specific RT-LAMP assay

**Figure 1:**
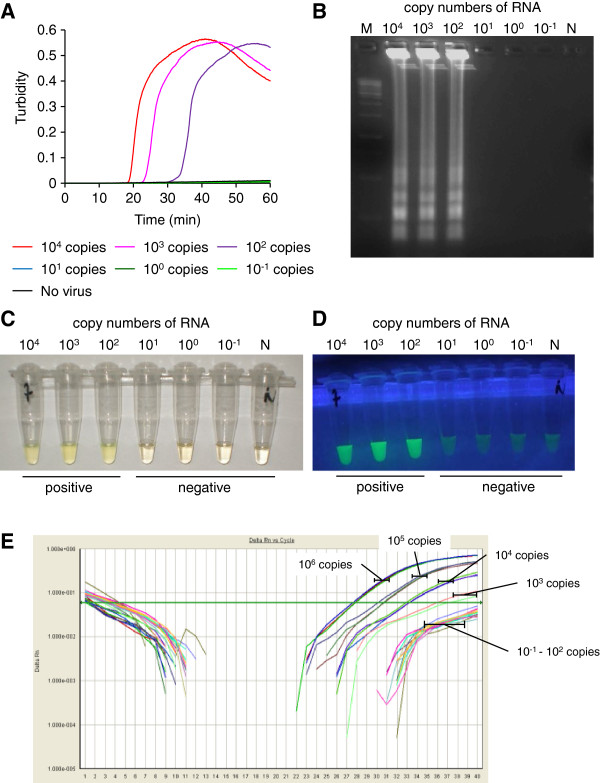
**Gene amplification and detection of the TBEV by TBEV-specific RT-LAMP assay.** (**A**) Real-time kinetics of the RT-LAMP amplification was monitored by real-time turbidimeter. *In vitro* transcribed RNA of TBEV Oshima strain was serially diluted to make 10^-1^ to 10^4^ copies. The reaction was repeated three times and a representative result is shown. (**B**) Agarose gel electrophoresis profile of the RT-LAMP products. M indicates 100-bp DNA marker (Sigma). N indicates a sample containing no viral RNA. (**C** and **D**) Visual detection of the RT-LAMP products amplified in a reaction tube. Positive amplification is indicated by change of color to cloudy yellow (**C**) or by fluorescent green under UV irradiation (**D**). (**E**) Real-time kinetics of TaqMan rRT-PCR with TBEV-specific primer/probe set. *In vitro* transcribed RNA of Oshima was serially diluted to make 10^-1^ to 10^6^ copies. The reaction was repeated three times and a representative result is shown.

The sensitivity of TBEV-specific RT-LAMP assay that targets the consensus sequence in NS1 gene of all TBEV subtypes was examined by using 10-fold serial dilutions of *in vitro* transcribed RNA of the FE-subtype Oshima strain (Figure 1A). The detection limit was 10^2^ copies of target RNA per reaction volume. All positive amplifications were achieved in less than 50 min. Agarose gel electrophoresis of the RT-LAMP products showed the typical ladder-like pattern of bands (Figure 1B). The amplified products were confirmed to have the expected nucleotide sequences (data not shown). Change of color to cloudy yellow was observed directly by the naked eye (Figure 1C) and fluorescent detection was observed under UV irradiation (Figure 1D) in samples with at least 10^2^ copies. On the other hand, the detection limit of the TaqMan rRT-PCR assay was 10^3^ copies of target RNA per reaction volume. Thus, our TBEV-specific RT-LAMP assay was more sensitive for the transcribed RNA of the Oshima strain than the rRT-PCR assay with TBEV-specific primers reported previously [14].

### TBEV-subtype specific RT-LAMP assay

**Figure 2:**
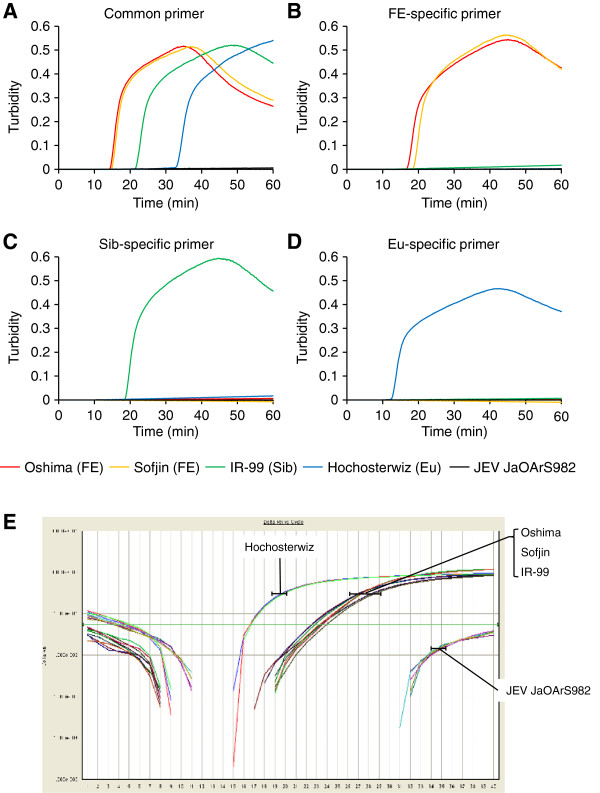
**Gene amplification and detection of different TBEV subtypes by subtype-specific RT-LAMP assay.** Real-time kinetics was monitored by the turbidity of RT-LAMP products after using 10 ng of RNA samples extracted from supernatants of cultured cells infected with the Oshima (FE), Sofjin (FE), IR-99 (Sib) and Hochostervitz (Eu) strains and JEV JaOArS982 strain. Each viral gene was amplified by (**A**) TBEV-specific RT-LAMP assay, (**B**) TBEV FE subtype-specific RT-LAMP assay, (**C**) TBEV Sib subtype-specific RT-LAMP assay, or (**D**) TBEV EU subtype-specific RT-LAMP assay. Each reaction was repeated three times and a representative result is shown for each sample. (**E**) Real-time kinetics of TaqMan rRT-PCR with TBEV-specific primer/probe set using 10 ng of RNA samples extracted from supernatants of cultured cells infected with the Oshima (FE), Sofjin (FE), IR-99 (Sib) and Hochostervitz (Eu) strains and JEV JaOArS982 strain. The reaction was repeated three times and a representative result is shown.

TBEV-specific RT-LAMP assay could clearly detect viral gene amplifications for the representative strain (s) of all subtypes of TBEV used in this study such as Oshima and Sofjin (FE), IR-99 (Sib) and Hochosterwitz (Eu), but not for JaOArS982 strain of Japanese encephalitis virus (JEV) (Figure 2A). All the positive amplifications were achieved in less than 50 min.

On the other hand, FE-specific RT-LAMP assay could amplify viral genes only of Oshima and Sofjin strains but not of IR-99 and Hochosterwitz strains, and JEV (Figure 2B). Sib- and Eu-specific RT-LAMP assay could amplify viral genes only of IR-99 and Hochosterwitz strains, respectively, but not of the other subtype strains and JEV (Figures 2C and 2D). Each subtype-specific RT-LAMP assay exhibited either similar or shorter reaction time for positive amplification when compared with TBEV-specific RT-LAMP assay done in the same RNA samples (Figure 2). These observations indicate that the sensitivity of each subtypes-specific RT-LAMP assay was either similar or higher compared with TBEV-specific RT-LAMP assay.

rRT-PCR assay using TBEV-specific primers could clearly detect viral gene amplifications for all subtypes of TBEV with the Hochosterwitz showing early amplification than the other strains (Figure 2E). These results indicate that our RT-LAMP assay can specifically amplify the target RNA of all TBEV subtypes as the rRT-PCR assay established previously [14].

### Evaluation of TBEV-specific RT-LAMP assay for amplification of gene from TBEV mixed with tick homogenates

**Figure 3:**
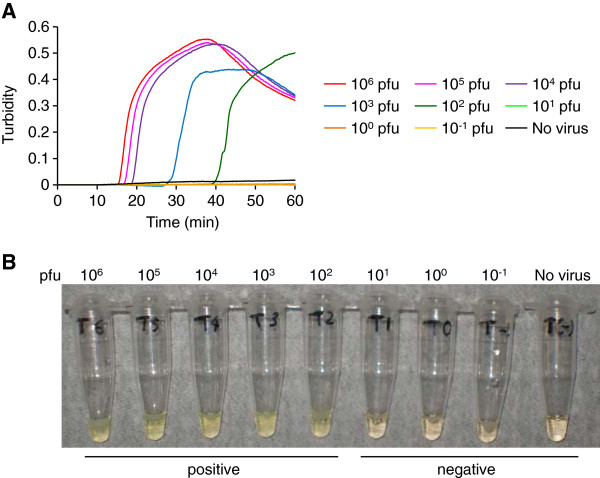
**Detection of TBEV gene from a mixture of TBEV and tick homogenates by TBEV RT-LAMP assay.** (**A**) Real-time kinetics monitored by the turbidity of RT-LAMP products after using 10 ng of RNA extracted from each mixture of TBEV and pooled tick homogenates. (**B**) Visual detection of color change to cloudy yellow in the RT-LAMP products. The reaction was repeated three times and a representative result is shown. RNA was extracted from 10 mg of ticks containing TBEV Oshima strain of different concentrations between 10^-1^ to 10^6^ pfu. Approximately 10 μg of RNA was recovered from one sample.

Next, we examined the possibility of using the TBEV-specific RT-LAMP assay for epidemiological study. To confirm the detection of TBEV gene in tick samples, we mixed TBEV Oshima strain prepared at different dilutions (10^-1^ to 10^6^ pfu) with pooled tick homogenates and extracted RNA from these mixtures. TBEV-specific RT-LAMP assay could amplify specific TBEV gene in the RNA extracted from tick samples with more than 10^2^ pfu of TBEV/10 mg of ticks (Figure 3A). Change of color to cloudy yellow was visualized in positive samples, but not in control sample containing no virus (Figure 3B). These results suggest that tick extract did not inhibit the amplification of viral gene in the assay.

### Evaluation of TBEV-specific RT-LAMP assay for amplification of gene from TBEV mixed with human blood

**Figure 4:**
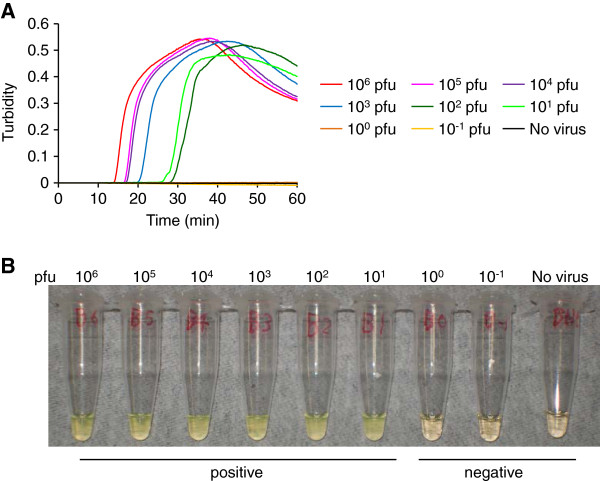
**Detection of TBEV gene from a mixture of TBEV and human blood by TBEV RT-LAMP assay.** (**A**) Real-time kinetics monitored by the turbidity of RT-LAMP products after using 10 ng of RNA extracted from each mixture of TBEV and human blood sample. (**B**) Visual detection of color change to cloudy yellow in the RT-LAMP products. The reaction was repeated three times and a representative result is shown. RNA was extracted from 200 μl of blood containing TBEV Oshima strain of different concentrations between 10^-1^ to 10^6^ pfu. Approximately 2 μg of RNA was recovered from one sample.

To show the possibility of using the TBEV-specific RT-LAMP assay for clinical diagnosis, we tried to detect the viral RNA of TBEV Oshima strain mixed in human blood samples. TBEV-specific RT-LAMP assay could amplify TBEV gene in the RNA samples extracted from human blood samples containing more than 10^1^ pfu of TBEV/200 μl of blood (Figure 4A). Color changed to cloudy yellow in positive samples, but not in control sample containing no virus (Figure 4B). These results suggest that blood extract did not inhibit the amplification of viral gene during the assay.

### Evaluation of TBEV-specific RT-LAMP assay for detection of TBEV gene in TBEV-infected mouse tissues

**Figure 5:**
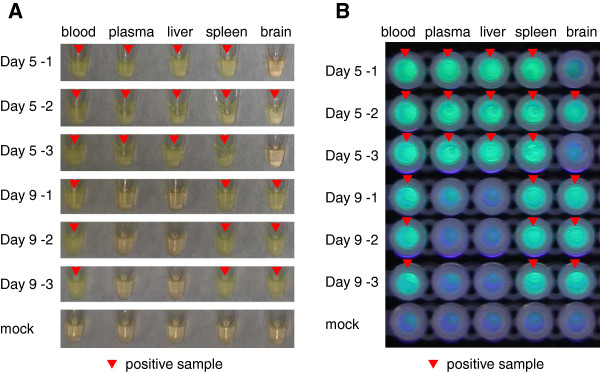
**Detection of TBEV gene from mouse tissue samples by TBEV RT-LAMP assay.** Visual detection of RT-LAMP products using 10 ng of RNA samples extracted from blood, plasma, liver, spleen and brain samples collected from laboratory mice on the 5th and 9th day following peripheral infections with TBEV Oshima strain. Three mice in each group and one mouse for mock infected mice were used. Positive amplification was indicated by the change of the color to cloudy yellow (**A**) or fluorescent green under UV irradiation (**B**). The reaction was repeated three times for each sample and a representative result is shown.

To show the possibility of applying TBEV-specific RT-LAMP assay for clinical samples, i.e. biopsies samples, we further attempted to detect the viral RNA in the tissues of laboratory mice infected with TBEV. As we previously showed, infectious viruses could be detected in the peripheral tissues 1 to 7 days post-infection (pi) and in the central nervous system (CNS) 5 days pi [27]. Thus, we collected blood, plasma, liver, spleen and brain samples of mice infected with the Oshima strain of TBEV on the 5th and 9th days pi. TBEV-specific RT-LAMP assay could amplify TBEV RNA in the peripheral tissues (blood, plasma, liver and spleen) of all mice and in the brain of one mouse at 5 days pi (Figure 5A and 5B). On the 9th days pi, all mice were positive for TBEV RNA in their blood and brain. Color change was not visualized in samples from mock-infected mice at 9 days pi (Figure 5A and 5B). These results closely reflected the time course of virus replication *in vivo* as reported previously [27].

## Discussion

This is the first report of an RT-LAMP assay for detection of TBEV gene and for differentiating all the three subtypes (FE, Sib and Eu) of TBEV from each other. We also raised the possibility that this assay will be useful for the detection of viral RNA from tick and clinical samples.

Over the last decades, TBE endemic areas and human cases have increased [7,28], although the disease severity caused by different subtype of TBEV may vary [3,7,8]. Co-circulation of the different subtypes exists in some countries, e.g. Latvia [29]. Therefore, investigation of the distribution of different subtypes in the field is important for epidemiological studies. In Europe, prevalence of TBEV in ticks is determined by identifying viral RNA through RT-PCR or rRT-PCR [10,11,30]. However, the first report on the use of real-time rRT-PCR [15] could not differentiate between subtypes. To differentiate TBEV subtype, some techniques have been developed, e.g. multiplex RT-PCR and pyrosequencing technique combined with rRT-PCR method [14,17]. However, PCR-based method has its own disadvantages due to the high cost of equipment and the requirement for highly skilled personnel. In this study, we showed that the TBEV subtype-specific RT-LAMP assay could differentiate subtypes with high sensitivity. The positive amplification could be confirmed by simple and direct visualization without the use of any expensive equipment. Furthermore, we also showed that tick extract did not inhibit the quality of the assay. Therefore, this assay might be suitable for the epidemiological study of TBEV and in field work.

For current laboratory diagnosis of TBE, serological assays are usually used [31,32]. In particular, IgM capture ELISA has been widely used for rapid diagnosis [33], because IgM antibody is detected in serum or CSF in the early phase of disease. However, IgM antibody appears as late as about two weeks after infection, and can persist for up to 10 months after vaccination or natural infection of a person [28]. Viral RNA is detectable before appearance of IgM antibody. Therefore, PCR-based TBEV diagnosis is an important alternative to serological methods in the early phase of the disease [15,19].

Owing to the simple and rapid performance of TBEV RT-LAMP assay, it has advantages over IgM capture ELISA or PCR-based TBEV diagnosis. Here, we showed that TBEV RT-LAMP assay could detect virus RNA from peripheral and CNS tissues following TBEV infection and was not inhibited by blood. Therefore, the assay we developed in this study will be useful for timely diagnosis and rapid laboratory confirmation of TBE suspected case-patients in TBE endemic countries especially in areas with resource-limiting condition.

## Conclusion

In conclusion, the TBEV RT-LAMP assay offers a sensitive, specific, rapid and easy-to-handle method for the detection of TBEV RNA in clinical samples obtained from patients during the first phase of their illness or in tick samples. We hope that this new technique will contribute to practical molecular diagnostic tool as well as in epidemiological surveillance.

## Methods

### Design of RT-LAMP assay primers for TBEV

A set of six primers comprising two outer primers (forward outer primer F3 and backward outer primer B3), two inner primers (forward inner primer FIP and backward inner primer LB), and two loop primers (forward loop primer FP and backward loop primer BP), were designed for the LAMP assay by using a software PrimerExplorer (version 4; Eiken Chemical Co., Ltd. Tokyo, Japan). The TBEV-specific primers were designed to be specific for NS1 gene based on the consensus sequence identified in 47 strains of TBEV. On the other hand, the TBEV subtype-specific primers were designed to be specific to E gene based on each consensus sequence identified in 63, 96 and 182 strains for FE, Sib and Eu subtypes, respectively. All primers were synthesized by Genenet Co., Japan. The details for each primer and the location in the genomic sequences are shown in the Table 1.

**Table 1:** Primer sets designed for RT-LAMP assay detection of TBEV in this study

**Primer**	**Sequence**	Genome position*	**Length (bp)**
TBEV-F3	ACCATAAATGCCGACTGTGA	3319–3338	20
TBEV-B3	TGCCACCACCATTGAGC	3500–3516	17
TBEV-FIP	CGGCAGCACCACTCTGGAATTCGGGGGCTTCTGTGAGGA	3345–3361	39
		3385–3404	
TBEV-BIP	TGCACACTACCTCCAGTGACGTGAATTCATGAACAGGACGTATTTCC	3409–3430	47
		3462–3480	
TBEV-LF	ACCTTGCCACTCTCTGTGG	3365–3383	19
TBEV-LB	ACGGGGACAGACTGTTGGTATGC	3436–3485	23
FE-F3	TTGACCTTGCTCAGACTGTC	1547–1566	20
FE-B3	CGTCCATTTTCACAGCGTGT	1713–1732	20
FE-FIP	TGAACCAGTCCCGGTGGACCTTGGAGCTTGACAAGACCTC	1569–1589	40
		1615–1633	
FE-BIP	GCCCTGCCGTGGAAACATGAGGAGCTCCAAACTCAACCA	1642–1663	39
		1694–1710	
FE-LF	CAGGCCGTCGGTAGGTGTTC	1591–1610	20
FE-LB	GGGAGCACAAAACTGGAACA	1662–1681	20
Sib-F3	CCACTCTGGCTGAAGAACAT	1211–1230	20
Sib-B3	ATTGGCGGCAACGTAGTC	1417–1434	18
Sib-FIP	GCCCTTTCCAAACAGTCCGCAAGCACGGTGTGCAAGAGAG	1240–1255	40
		1285–1305	
Sib-BIP	TGAGGCAAAGAAGAAGGCCACTCGTGTGTGGCTCAACCTTA	1335–1356	41
		1395–1413	
Sib-LF	CCCAGCCTCGATCACTCTGGT	1256–1276	21
Sib-LB	GGACATGTGTATGACGCCAACAAG	1357–1380	24
EU-F3	CGCAAAACTGGAATAACGCA	1667–1686	20
EU-B3	CCACTTCGCAGGTCACATG	1831–1849	19
EU-FIP	ACTCCAGTCTGGTCTCCGAGGACTGGTTGAATTTGGGGCTC	1691–1711	41
		1741–1760	
EU-BIP	GTTACTGAAGGCTCTCGCTGGGGCCACTCTTCAGGTGGTACT	1761–1783	42
		1811–1829	
EU-LF	ACACGTCCATCTTGACAGCGT	1715–1735	21
EU-LB	GTTCCTGTGGCACACATTGA	1783–1802	20

*genome position is based on the sequence of Neudoerf strain (accession no. TEU27495).

### Cell and virus

Stocks of the Oshima (FE) [34], Sofjin (FE), IR-99 (Sib) [5] and Hochosterwitz (Eu) strains of TBEV, and JaOArS982 strain of JEV were prepared in baby hamster kidney (BHK) cells [5,34]. Hochosterwitz strain was kindly provided by F.X. Heinz (Medical University of Vienna), and the supernatant of infected BHK cells was kindly provided by K. Yoshii, (Hokkaido University). Virus titers were determined by plaque forming assays done in BHK cells and were expressed as pfu/ml [27,35]. The BHK cells were maintained in Eagle’s Minimal Essential Medium (EMEM; Nissui Pharmaceutical Co.) containing 10% fetal calf serum (FCS). All experiments using live TBEV were performed in a biosafety level 3 laboratory of the Institute of Tropical Medicine, Nagasaki University according to standard BSL3 guidelines.

### Mice

B6 mice were purchased from Japan SLC Corporation. Five week old female mice were subcutaneously inoculated with 10^3^ pfu of the Oshima strain of TBEV diluted in EMEM containing 2% FCS. Mock infected mice were inoculated with the supernatant medium of uninfected BHK cells. At 5 and 9 days pi, blood, liver, spleen and brain samples were collected after euthanasia. Blood samples were centrifuged to obtain plasma. The animal experimental protocols were approved by the Animal Care and Use Committee of the Nagasaki University (approval number: 100723–1/1008050873).

### *In vitro* transcription and quantification

To obtain a quantitative RNA standard with which to examine the detection limit of RT-LAMP assay, a plasmid containing full length sequence of the Oshima strain of TBEV was used for RNA transcription [36]. The plasmid was digested with Not I and Sma I (Takara) for 1 hour and the fragment was purified with QIAquick Gel Extraction Kit (Qiagen). The number of DNA copies/μl was calculated based on a previous report [37]. The DNA fragment was then transcribed *in vitro* with SP6 transcriptase and digested with DNAse by using mMESSAGE mMACHINE (Ambion) according to the manufacturer’s protocol. The RNA was purified with an RNeasy minikit (Qiagen). The number of RNA copies/μl was determined by using the following formula: concentration of RNA (g/μl)/[(transcript length in nucleotides × 340)] × 6.022 × 10^23^, where the RNA length is 4966 base. The RNA concentration was measured with a NanoDrop ND-1000 apparatus (Thermo Scientific). The target RNA copy number was calculated and serial dilutions ranging from 10^-1^ to 10^4^ RNA copies were used to determine the range of quantification.

### RNA extraction

A 250 μl volume of culture supernatant from infected BHK cells was mixed with 750 μl of Isogen-LS (Nippon Gene) and genomic viral RNA was extracted according to the manufacturer’s protocol. Ten-fold serial dilutions (10^-1^ to 10^6^ pfu) of the Oshima strain of TBEV were prepared. Each diluted sample was added to 10 mg of pooled ticks (*Ixodes persulcatus*, laboratory breeding ticks was kindly provided by S. Konnai, Hokkaido University) and homogenized in 1 ml of Isogen (Nippon Gene). A 200 μl volume of healthy human blood was mixed with 50 μl of the Oshima strain of TBEV prepared at different concentrations (10^-1^ to 10^6^ pfu) and with 750 μl of Isogen-LS. RNA was extracted according to the manufacturer’s protocol. Total RNAs of mouse blood and plasma samples were extracted using Isogen-LS (Nippon Gene). Total RNAs of mouse livers, spleens and brains were extracted using RNeasy Lipid Tissue Mini Kit (Qiagen) according to the manufacturer’s protocol. RNA was dissolved in DPEC-treated water and stored at −80°C until use. The experiment using human blood was performed with the approval of ethics committee of the Institute of Tropical Medicine, Nagasaki University (approval number: 121226102).

### RT-LAMP

RT-LAMP was performed in a final reaction volume of 25 μl using a Loopamp RNA amplification kit (Eiken Chemical Co., Ltd. Tokyo, Japan) with 5 pmol of the primers F3 and B3, 20 pmol of the primers LF and LB, and 40 pmol of the primers FIP and BIP. One microliter of the extracted RNA was used as template per reaction. The RT-LAMP reaction mixtures were incubated at 62.5°C for 60 min and inactivated at 80°C for 5 min.

### Analysis of RT-LAMP product

A real-time turbidity caused by the accumulation of magnesium polyphosphate was monitored spectrophotometrically at 650 nm with LA-320C Loopamp real-time turbidimeter (Teramecs, Japan). The results were analyzed by the LA-320C software package (Teramecs). The product was electrophoresed on an agarose gel in Tris-acetate-EDTA (TAE) buffer followed by staining with ethidium bromide. Amplified products were sequenced to show that they matched the expected nucleotide sequences. Positive amplification was shown by the specific ladder-like pattern on a UV transilluminator at 320 nm. For visualization of the positive reaction, fluorescent detection reagent (FD; Eiken Chemical Co., Ltd. Tokyo, Japan) was added to the reaction mixture and a change of color was recognized directly (transparent to cloudy yellow color) or under UV irradiation (fluorescent green).

### rRT-PCR

One step rRT-PCR was done with One Step PrimeScript® RT-PCR Kit (TAKARA BIO INC.) according to the manufacturer’s instructions. TBEV-specific primers and probe that detect all TBEV subtypes described in the previous paper [14] were synthesized by Genenet Co., Japan. Hydrolysis minor groove binder probe (ABI PRISM® TaqMan® MGB probe) was obtained from Applied Biosystems. The rRT-PCR assay was performed with an Applied Biosystems 7500 rPCR system. One microliter of the extracted RNA was used as the template in each reaction mixture. A sample with the growth curve crossing the threshold line within 40 cycles was considered positive.

## Competing interests

The authors declared they have no competing interests.

## Authors’ contributions

DH designed the experiment, performed live virus infection, extracted RNA, did RT-LAMP assay, and wrote the manuscript. KA designed the primers for RT-LAMP assay, extracted RNA, and carried out RT-LAMP assay and rRT-PCR. KM participated in discussion of the results and helped in drafting the manuscript. All authors read and approved the final manuscript.
